# Prognostic value of myocardial work indices measured on echocardiography in patients with aortic stenosis undergoing transcatheter aortic valve replacement: A systematic review and meta-analysis

**DOI:** 10.12669/pjms.42.4.15217

**Published:** 2026-04

**Authors:** Juan Zhou, Ronghua Liu, Fei Chen, Yujia Chen, Qinyu Xiao, Yuehui Yu

**Affiliations:** 1 Juan Zhou, Department of Ultrasound, The First Hospital of Jiaxing, Jiaxing, Zhejiang Province 314001, P.R. China; 2 Ronghua Liu, Department of Ultrasound, The First Hospital of Jiaxing, Jiaxing, Zhejiang Province 314001, P.R. China; 3 Fei Chen, Department of Ultrasound, The First Hospital of Jiaxing, Jiaxing, Zhejiang Province 314001, P.R. China; 4 Yujia Chen, Department of Ultrasound, The First Hospital of Jiaxing, Jiaxing, Zhejiang Province 314001, P.R. China; 5 Qinyu Xiao, Department of Ultrasound, The First Hospital of Jiaxing, Jiaxing, Zhejiang Province 314001, P.R. China; 6 Yuehui Yu, Department of Ultrasound, The First Hospital of Jiaxing, Jiaxing, Zhejiang Province 314001, P.R. China

**Keywords:** Aortic stenosis, Myocardial work indices, Mortality, TAVR

## Abstract

**Background & Objective:**

Myocardial work (MW) indices offer an afterload-adjusted assessment of left ventricular (LV) function and may improve risk stratification in patients with severe aortic stenosis (AS) undergoing transcatheter aortic valve replacement (TAVR). This systematic review and meta-analysis aimed to evaluate the prognostic significance of MW indices, global work index (GWI), global constructive work (GCW), global wasted work (GWW), and global work efficiency (GWE), in patients with severe AS undergoing TAVR.

**Methodology:**

PubMed, Embase, Web of Science, and Scopus were searched from inception to 26 November 2025 for studies assessing prognostic value of MW indices in TAVR-treated AS patients. Data were synthesised qualitatively, and a random-effects meta-analysis was performed when at least three studies reported comparable effect estimates.

**Results:**

Five cohort studies met the inclusion criteria. Across studies, GWI consistently demonstrated strong prognostic performance. Lower post-TAVR GWI predicted mortality in multiple cohorts, with thresholds ranging from 1,095 to 1,234 mmHg% and area-under-the-curve values up to 0.71. Pooled analysis confirmed that higher GWI was associated with reduced mortality (HR: 0.94 95% CI: 0.90, 0.98 I^2^=68% p=0.004). GCW showed modest prognostic value, whereas GWW was not independently predictive. GWE was a significant predictor in select studies, but mostly in univariate models.

**Conclusion:**

Limited evidence shows that MW indices, especially GWI, may provide meaningful prognostic information in AS patients undergoing TAVR. Further studies are needed to improve the evidence.

***Registration No.:*** PROSPERO database (CRD420251238182).

## INTRODUCTION

Aortic stenosis (AS) is the most prevalent valvular condition among older individuals, marked by a progressive elevation in left ventricular (LV) pressure overload.^1^ As a result, the LV undergoes compensatory hypertrophic remodelling, which, if not addressed, can progress to subclinical or overt systolic dysfunction and heart failure symptoms.^2^ Transcatheter aortic valve replacement (TAVR) is increasingly being adopted as a widely accepted procedure for the management of severe AS or for patients who are asymptomatic yet exhibit an impaired left ventricular ejection fraction (LVEF).^3^ It greatly increases survival with minimal morbidity.^4^,^5^ However, many patients do not achieve meaningful improvement in LV function after valve replacement, leading to persistent symptoms and poor long-term outcomes.^2^ This underscores the need to improve methodologies for evaluating cardiac function and to refine risk categorisation both prior to and following TAVR.^6^

Historically, LVEF has served as a metric for assessing systolic function; however, it frequently remains intact until the later stages of AS and is significantly affected by loading conditions.^2^ Global longitudinal strain (GLS) exhibits heightened sensitivity; however, it fails to account for the elevated afterload associated with severe AS.^7^ These challenges complicate the differentiation between genuine cardiac dysfunction and load-dependent functional impairment, particularly across the several physiological subtypes of AS.^8^ Given the rising prevalence of TAVR in elderly patients with various health complications, the demand for more dependable prognostic and recovery indicators has intensified.

Myocardial work (MW) indices, obtained through pressure-strain loop analysis that integrates strain imaging with a non-invasive estimation of LV systolic pressure, have gained recognition as a promising method to bridge existing diagnostic gaps.^9^ By accounting for afterload in the evaluation of myocardial deformation, parameters such as global work index (GWI), global constructive work (GCW), global wasted work (GWW), and global work efficiency (GWE) offer a more comprehensive assessment of LV function than strain measurements alone.^10^ Validated techniques for estimating LV pressure in patients with severe AS now enable reliable application of MW measurement within this cohort. Preliminary research indicates that MW correlates with symptom severity, myocardial injury, and adverse clinical outcomes, and may provide superior prognostic value compared to traditional echocardiographic markers, particularly in identifying high-risk patients undergoing TAVR.^10^-^12^ Despite these developments, the evidence remains scattered, and no comprehensive review has specifically assessed the prognostic value of MW indices in AS patients undergoing TAVR. Therefore, a systematic review and meta-analysis are needed to compile existing data, measure the prognostic relevance, and determine the clinical usefulness of these emerging parameters.

## METHODOLOGY

A comprehensive, multi-database search was carried out on PubMed, Embase, Web of Science, and Scopus from inception until 26^th^ November 2025 using a combination of controlled vocabulary and free-text keywords related to aortic stenosis (“aortic stenosis,” “severe AS”), transcatheter aortic valve replacement (“TAVR,” “TAVI,” “transcatheter aortic valve implantation”), and MW indices (“myocardial work,” “global work index,” “GWI,” “global constructive work,” “GCW,” “global wasted work,” “GWW,” “global work efficiency,” “GWE,” and “pressure–strain loop”). We did not include any keywords related to outcomes, as the search results were very few. Instead, we manually screened all records pertaining to MW indices and AS to extract all studies reporting relevant outcomes (Supplementary Table-I). Two reviewers (JW & RL) independently performed the literature search. To ensure completeness, all reference lists of included studies and relevant reviews were manually reviewed. We also ran a gray literature search using the database of Google Scholar for any missed articles. The review has been reported according to PRISMA guidelines,^13^ with the protocol uploaded to the PROSPERO database (CRD420251238182).

**Supplementary Table-I:** Search strategy.

***PubMed***
(“aortic stenosis”[MeSH Terms] OR “aortic stenosis” OR “severe aortic stenosis” OR “AS”) AND (“transcatheter aortic valve replacement”[MeSH Terms] OR “transcatheter aortic valve replacement” OR “transcatheter aortic valve implantation” OR “TAVR” OR “TAVI”) AND (“myocardial work” OR “global work index” OR “GWI” OR “global constructive work” OR “GCW” OR “global wasted work” OR “GWW” OR “global work efficiency” OR “GWE” OR “pressure–strain loop” OR “pressure strain loop”)
***Embase***
('aortic stenosis'/exp OR 'aortic stenosis':ti,ab OR 'severe aortic stenosis':ti,ab) AND ('transcatheter aortic valve replacement'/exp OR 'TAVR':ti,ab OR 'TAVI':ti,ab OR 'transcatheter aortic valve implantation':ti,ab) AND ('myocardial work':ti,ab OR 'global work index':ti,ab OR GWI:ti,ab OR 'pressure strain loop':ti,ab OR 'constructive work':ti,ab OR 'wasted work':ti,ab OR 'work efficiency':ti,ab)
***Web of Science***
TS = ((“aortic stenosis” OR “severe aortic stenosis”) AND (“transcatheter aortic valve replacement” OR “transcatheter aortic valve implantation” OR TAVR OR TAVI) AND (“myocardial work” OR “global work index” OR GWI OR GCW OR GWW OR GWE OR “pressure strain loop”))
***Scopus***
(TITLE-ABS-KEY(“aortic stenosis” OR “severe aortic stenosis”) AND TITLE-ABS-KEY(“transcatheter aortic valve replacement” OR “transcatheter aortic valve implantation” OR TAVR OR TAVI) AND TITLE-ABS-KEY(“myocardial work” OR “global work index” OR GWI OR GCW OR GWW OR GWE OR “pressure strain loop”)).

**Supplementary Table-II:** GRADE assessment of evidence.

	GWI and mortality
Number of studies	3
***Downgrade quality of evidence***	
Risk of bias	No
Inconsistency	No
Indirectness	No
Imprecision	Very serious*
***Publication bias***	
***Upgrade quality of evidence***	
Large effect	No
Plausible confounding	No
Dose-response	No
Overall certainty of Evidence	Very low

* small number of studies in the meta-analysis

Studies were eligible if they involved adult patients with moderate-to-severe or severe AS undergoing TAVR. They had to assess at least one MW parameter using non-invasive pressure–strain loop–based echocardiography (such as GWI, GCW, GWW, or GWE) and report prognostic outcomes like all-cause mortality, cardiovascular death, or combined endpoints such as death plus heart failure hospitalisation. To allow both qualitative and quantitative synthesis, we also included studies that did not provide effect ratios for the above outcomes. We also chose to include studies reporting post-TAVR MW indices rather than pre-TAVR indices to maintain homogeneity across the included studies.

### Exclusion criteria included:

- Studies focusing solely on LV strain without MV indices.
- Review articles.
- Editorials.
- Duplicate publications. If two studies used the same dataset, we chose to include the study which included more outcome variables or MW indices and was more coherent with the inclusion criteria.

Search results were imported into EndNote software, and duplicate entries were electronically eliminated. Three authors (FC, YC, QX) independently screened all titles and abstracts of relevant papers, applying predefined inclusion and exclusion criteria. Throughout this process, any articles with uncertain inclusion or exclusion status were highlighted, and the three authors convened to discuss and reach consensus. The same three authors independently reviewed the full texts of the selected articles, followed by discussions to achieve agreement on their inclusion.

Data extraction was independently conducted by two reviewers using a standardised data collection Form (FC & YY). Extracted variables included study characteristics (first author, publication year, country, study design, and sample size), patient demographics (gender, comorbidities) and baseline clinical features (Euro score, LVEF), the specific MW indices measured (GWI, GCW, GWW, GWE), timing of echocardiography post TAVR, follow-up duration, outcomes, study results and fully adjusted or unadjusted effect estimates of the outcomes for each MW index. If three studies were available for a similar outcome, a quantitative synthesis was conducted.

Quality assessment was based on the Newcastle–Ottawa Scale (NOS).^14^ The scale has multiple questions that evaluate the selection of study populations, the comparability of cohorts, and the adequacy of outcome assessment and follow-up. The maximum score for each of these three domains is four, two and three, respectively. Studies scoring ≥7 were classified as high quality. Two reviewers (YC & QX) were independently involved in the quality assessment process, with disagreements being resolved by involving the third reviewer (YY).

The majority of the data was qualitatively analyzed. For quantitative pooling, effect measures were synthesized using a random-effects model. Review Manager (version 5.3) was utilized for this purpose. Heterogeneity was assessed using the I² statistic. Publication bias was not examined due to limited data. Sensitivity analysis was conducted by removing one study at a time. GRADE approach was used to assess the certainty of evidence.

## RESULTS

**Fig.1:**
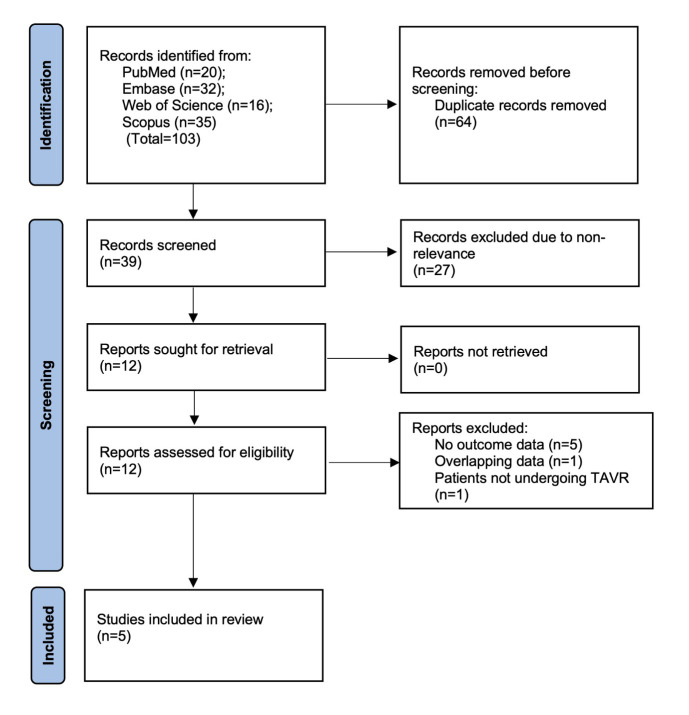
Study flowchart.

A total of 103 studies were found across the four databases. No additional study was identified from gray literature or reference list searching. After removing 64 duplicates, 39 unique studies remained for title and abstract screening. Of these, 27 were deemed irrelevant and excluded. Full texts of the remaining 12 articles were then retrieved for detailed eligibility assessment, resulting in the exclusion of six studies: five due to missing relevant outcome data, one due to overlapping populations, and one for not including patients treated with TAVR. Five studies^11^,^15^-^18^ met all inclusion criteria, as illustrated in the PRISMA flow diagram (Fig.1).

The five included studies were conducted between the years 2020 and 2024 across Switzerland, Italy, Belgium, Denmark, and the Netherlands (Table-I). The studies included 1,070 patients with moderate-to-severe or severe AS undergoing TAVR. Two studies were prospective, and three were retrospective cohort studies. The median or mean age of patients ranged from 79 to 83 years. Baseline cardiovascular comorbidity burdens were consistently high across cohorts, with hypertension present in 74–91% of patients and coronary artery disease in 33–55%. Diabetes mellitus was reported in 21–28% of participants in studies that reported it.

All studies incorporated MW indices for prognostic assessment, with GWI reported in all five studies. Four studies reported additional indices, including GCW, GWW, and GWE. The timing of echocardiographic assessment varied, ranging from immediately post-TAVR to one month after the procedure, while one study included serial assessments at both one day and one month. Follow-up durations were heterogeneous, ranging from 12 months to a median of 60–625 days across studies. Outcomes also differed among studies: two assessed all-cause mortality, one focused on cardiovascular mortality, and two reported composite endpoints combining mortality with heart failure–related events or symptom worsening. Mortality rates ranged widely, from 6% to 51%. On quality assessment, two studies scored nine, two studies scored eight and one study scored six.

### GWI:

All five studies identified GWI as a central and powerful prognostic marker. In Anwer et al.,^15^ lower GWI measured within two weeks after TAVR strongly predicted cardiovascular mortality, with non-survivors showing significantly reduced GWI (median 1,029 mmHg% [IQR 641–1,382] vs 1,389 mmHg% [IQR 1,070–1,653], p=0.003). In univariable Cox regression, GWI was significantly associated with cardiovascular mortality (p=0.004), and a cut-off of ≤1,234 mmHg% yielded an area under the curve (AUC) of 0.71. Survival was significantly worse using this threshold [hazard ratio (HR): 0.94 95% CI: 0.89, 0.98].

Ilardi et al.^11^ evaluated GWI at one month post-TAVR and noted that although GWI decreased after the procedure, lower values at follow-up were not associated with higher risk of their composite endpoint (death + Heart failure rehospitalization + dyspnea worsening) (HR: 0.999 95% CI: 0.998, 1.003). GWI was found to be non-significant in univariable analyses, and not included in multivariable models.

Moya et al.^16^ demonstrated that baseline GWI was markedly lower among patients who later developed the composite outcome (mortality + heart failure hospitalization). They identified a baseline cut-off of 2,323 mmHg% with good discriminative performance (sensitivity 63%, specificity 76%), and adding GWI to other variables improved the performance of prognostic model. Their study, however, failed to report HRs for GWI.

In the largest cohort, Pedersen et al.^17^ noted that, pre-TAVI GWI was independently associated with all-cause mortality over a median 60-month follow-up. Each 100 mmHg% increase in baseline GWI corresponded to a 4% reduction in mortality risk (HR 0.96, 95% CI 0.92–1.00, p=0.033). Similar results were observed at one-month follow-up, where GWI remained independently predictive even after adjusting for EuroSCORE II, GLS, and stroke volume index (HR: 0.93, 95% CI 0.90, 0.96, p<0.001).

**Table-I:** Details of included studies.

Study/ Location	Design	Sample size	Age (years)	Males (%)	DM (%)	HT (%)	CAD (%)	Euro score	LVEF (%)	Work index	Timing of Echo	Follow-up	Outcome	Mortality rate (%)	NOS score
Anwer 2024 Switzerland	P	144	82 [78–86]	51	26	79	50	2.9 [1.9–5.0]	58 [52–64]	GWI, GWE	Within 2 weeks of TAVR	625 [511–770] days	Cardiovascular mortality	14	S-4 C-2 O-3
Ilardi 2024 Italy	R	88	79.9 ± 6.4	40	26	91	33	5.3 ± 6.0	58 ± 8.5	GWI, GWW, GWE	1 month post-TAVI	12 [12–23] months	Composite of death + HF rehospitalization + dyspnea worsening	6	S-4 C-1 O-3
Moya 2024 Belgium	P	110	83 ± 6	45	NR	NR	NR	NR	51 ± 10	GWI, GCW	1 day & 1 month after TAVR	521 ± 343 days	Composite (all-cause death + HF hospitalization)	20	S-4 C-0 O-2
Pedersen 2024 Denmark	R	473	80 ± 7	48	21	74	34	2.6 (1.7–4.5)	59 ± 6 (preserved LVEF group)	GWI	1 month post-TAVI	60 [45–69] months	All-cause mortality	NR	S-4 C-1 O-3
Wu 2024 Netherlands	R	255	82 [77–85]	51	28	69	55	NR	56 (45–63)	GWI, GCW, GWE, GWW	Immediately post-TAVR	59 [40–72] months	All-cause mortality	51	S-4 C-2 O-3

P, Prospective; R, Retrospective; DM, diabetes mellitus; HT, hypertension; CAD, coronary artery disease; GWI, Global work index; GCW, global constructive work; Echo, Echocardiography; TAVR, transcatheter aortic valve replacement; GWW, global wasted word; GWE, global work efficiency; NR, not reported; NOS, Newcastle Ottawa Scale; S, selection of studies; C, comparability; O, outcome assessment

**Fig.2:**
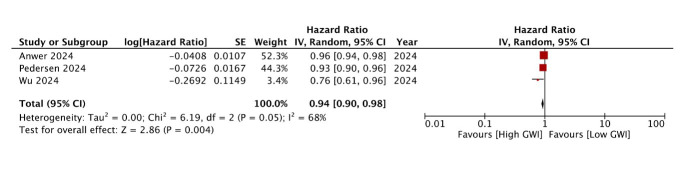
Meta-analysis of the association between post-TAVR GWI and mortality in AS patients.

Wu et al.^18^ reported that post-TAVR GWI was the strongest independent predictor of all-cause mortality among all LV function measures, outperforming post-TAVR GLS and GCW. In multivariable analysis, each tertile increase in post-TAVR GWI was associated with a 24% reduction in mortality (HR: 0.764, 95% CI 0.610, 0.956, p=0.019). The prognostic association persisted even after adjustment for pre-TAVR GWI. We would include three studies in the meta-analysis for GWI. Pooled analysis of three studies reporting adjusted HR showed that post-TAVR high GWI was a significant predictor of mortality (HR: 0.94 95% CI: 0.90, 0.98 I^2^=68% p=0.004) (Fig.2). No data conversion was needed for the meta-analysis. The results were not stable on sensitivity analysis and turned non-significant on exclusion of individual studies. GRADE assessment showed very low certainty of evicence.

### GCW:

GCW was less commonly studied in the included studies. Moya et al.^16^ noted that GCW was significantly lower in patients who developed the composite outcome (mortality + heart failure hospitalization) as compared to those who did not (2604 ± 913 vs 3061 ± 928, p=0.03). The AUC of GCW to predict adverse outcome was 0.657, however, their study failed to report sensitivity, specificity values as well as HR values for GCW. Wu et al.^18^ found that post-TAVR GCW independently predicted all-cause mortality (HR: 0.775, 95% CI: 0.620, 0.969, p=0.026), though it had a lower incremental prognostic gain than GWI.

### GWW:

GWW was the least consistent marker across studies. Ilardi et al.^23^ documented a significant early reduction in GWW after TAVR and a borderline association with adverse outcomes in univariable models (HR: 1.000, 95% CI: 1.000, 1.005), but GWW lost significance in multivariable analyses. Wu et al.^18^ likewise found no significant prognostic contribution of post-TAVR GWW (HR per tertile increase: 1.041, 95% CI: 0.842, 1.288 p=0.710).

### GWE:

Across studies, GWE emerged as an essential but inconsistent functional marker. Ilardi et al.^11^ identified post-TAVR GWE as the strongest independent predictor of their composite endpoint (HR: 0.905, 95% CI 0.832, 0.984 p=0.020). A GWE ≤ 92% identified a markedly high-risk group (29.5% vs 4.8% event rate at one year; p=0.003). Anwer et al.^15^ also reported lower GWE among non-survivors and a significant univariable association with cardiovascular mortality (HR: 0.94 95% CI: 0.89, 0.98). Wu et al.^18^ found that GWE decreased significantly after TAVR but was not associated with mortality in univariate models (HR per tertile increase: 0.871, 95% CI: 0.704, 1.077 p=0.203).

## DISCUSSION

In patients with severe AS undergoing TAVR, MW indices have emerged as valuable tools for evaluating LV performance and detecting residual myocardial impairment beyond traditional echocardiographic parameters.^10^,^12^ Conventional measures such as LVEF and GLS frequently fail to fully characterise the complex interactions between pressure overload, myocardial remodelling, and afterload modifications, as these parameters remain heavily load-dependent and may appear preserved despite underlying subclinical dysfunction.^7^,^19^ MW indices, which integrate strain with non-invasively estimated systolic pressure, offer a more physiologically based assessment of LV energetics.^20^ TAVR has been extensively shown to enhance symptoms, functional capacity, and survival rates in patients with severe AS.^4^,^21^

However, even after successful valve replacement and hemodynamic improvement, some residual myocardial damage may remain, potentially affecting long-term results. Evidence from mechanistic and clinical studies indicates that MW variables respond sensitively to the hemodynamic relief following TAVR. For example, Quinio et al.^22^ observed notable decreases in GWI and GCW of 28% to 33% immediately following TAVR, even though there were no significant changes in LVEF or GLS. Crucially, these declines were consistent across patient groups with normal or impaired baseline function, highlighting their reliability across the full range of AS severity. GWI and GCW are different measures of systolic function. GWI reflects the total work performed during systole, while GCW indicates the positive work of the myocardium during systolic shortening and isovolumic relaxation lengthening (GCW).^9^ Both parameters typically rise early in the disease because of increased afterload but then gradually decrease as fibrosis occurs and myocardial reserve diminishes.^23^,^24^ The decline in GWI and GCW after TAVR appears to be a normal response to decreased afterload and myocardial oxygen demand resulting from the removal of mechanical support obstruction.^9^

The broader evidence base is corroborated by a systematic review and meta-analysis conducted by Leo et al.,^25^ which aggregated data from 11 studies and confirmed statistically significant reductions in GWI (−236.7 mmHg%) and GCW (−243.7 mmHg%) following TAVR, with 95% CI of −373.8 to −99.5 and −407.4 to −80.0, respectively. Meta-regression analyses further demonstrated that age and baseline LVEF significantly modulate changes in GWI and GCW (p=0.041 and p=0.020), suggesting that myocardial efficiency post-TAVR is influenced by intrinsic myocardial reserve and the duration of pressure overload. Conversely, measures of GWW and GWE did not show consistent improvements, indicating persistent energetic inefficiency and residual myocardial damage in numerous patients despite hemodynamic correction. Overall, these findings demonstrate the utility of MW indices, particularly GWI and GCW, as sensitive markers of LV unloading, reverse remodelling, and ongoing myocardial impairment, which may offer prognostic insights beyond traditional echocardiographic assessments in post-TAVR patients.

Our systematic review builds upon this growing evidence to provide the first detailed analysis of the prognostic value of MW indices in the post-TAVR scenario. It was noted that GWI could be a meaningful prognostic factor in patients with severe AS undergoing TAVR. Across the included studies, lower GWI consistently identified patients at elevated risk of mortality or composite adverse events. In several cohorts, GWI also outperformed both GLS and LVEF in multivariable analyses.^15^,^17^ A small meta-analysis conducted in the review also reinforced the finding that low GWI is a significant predictor of mortality in AS patients undergoing TAVR.

Nevertheless, heterogeneity was substantial, and given the small number of studies, detailed subgroup or meta-regression could not be conducted. GCW was less commonly assessed and was lower in patients with adverse outcomes in one study. At the same time, another found it to be a significant independent predictor of adverse outcomes. However, its independent prognostic value was less consistent once GWI or GWE were considered. In our review, we also noted that GWE was inconsistently associated with post-TAVR outcomes. Only the study by Ilardi et al.^23^ found a statistically significant association, while two other studies showed no significant association. Likewise, GWW emerged as the least reliable marker, rarely retaining significance after adjustment. These results are consistent with those of Leo et al.,^25^ who reported significant reductions in GWI and GCW, reflecting the immediate hemodynamic unloading after TAVR. They also emphasized that these changes do not necessarily indicate full myocardial recovery, as GWE and GWW showed no consistent improvement.

MW indices have also been used to predict outcomes in cases of asymptomatic AS. In a study of 170 patients with asymptomatic moderate-to-severe AS and a LVEF of at least 50%, the Ilardi et al.^23^ have found that a GWI of 1951 mmHg% or less, along with a GCW of 2475 mmHg% or less, effectively predicted both all-cause and cardiovascular mortality over a four-year period. However, data on asymptomatic AS remains limited. One study^10^ also shows that MW indices may also not predict the need for TAVR in moderate AS and there is a need for further research on the prognostic ability of these indices in asymptomatic and moderate AS cases.

Limitations also exist regarding MW indices. A reduction in MW indices following TAVR has not been consistently observed in the literature. Myon et al.^26^ demonstrated that MW parameters did not show significant improvement after afterload reduction. The authors proposed several potential reasons, including delayed surgery in a myocardium already severely damaged or inadequate postoperative pressor management. Additionally, the development of conduction abnormalities and dyssynchrony can sometimes lead to incomplete reverse remodelling. Such conditions may therefore limit the prognostic ability of post-TAVR MW indices. MW analysis shares a similar limitation with GLS: it relies on good image quality to detect the endocardial border. Nonetheless, this limitation is minimal, as demonstrated by excellent agreement in both patients with heart disease and healthy individuals.^27^ Variability differs among the parameters. In evaluating interobserver variability, GWI and GCW have shown the highest pooled interobserver reliability, followed by GWW and GWE.^28^ Only further studies comparing the prognostic ability of GLS vis-à-vis MW indices can give more evidence on which parameter remains most valuable in predicting outcomes after TAVR.

### Limitations:

that should be recognised. First, all the included studies were observational cohort studies, which inherently entail confounding and limit causal inference. The quality of some of the studies was not high as they scored low in NOS. Low quality studies introduce bias in the evidence and the conclusions must be interpreted with caution. The studies showed significant heterogeneity due to differences in imaging protocols, timing of echocardiographic assessments (from immediately after TAVR to one month later), and variations in follow-up duration and clinical endpoints. MW indices were inconsistently reported across studies, and many lacked fully adjusted effect estimates, limiting comprehensive pooled analyses for GCW, GWW, and GWE. The cut-off values defining high-risk groups were varied or not reported at all in several studies. Additionally, publication bias cannot be ruled out, given the small number of studies and the likelihood that negative or neutral results are underreported. Lastly, all cohorts consisted of older, high-risk European populations, which restricts the applicability of the findings to younger patients, low-risk groups, or non-European regions. These limitations highlight the need for large, prospective, standardized studies to confirm the prognostic utility of MW indices in TAVR patients.

This review has notable strengths. It offers the first systematic review of the prognostic significance of MW indices specifically in patients with aortic stenosis undergoing TAVR. By systematically analyzing individual MW parameters rather than treating them as a single composite, this study clarifies the distinct prognostic value of GWI, GCW, GWE, and GWW, with GWI emerging as the most reliable and consistent marker. Clinically, these findings suggest that MW indices, especially the GWI, could enhance post-TAVR risk assessment beyond traditional measures such as LVEF and GLS. Since MW reflects myocardial deformation linked with afterload, it offers a more physiologically meaningful evaluation of ventricular function in pressure-overload conditions. Including MW assessment in routine echocardiography may help detect patients with ongoing energy deficiency despite successful valve placement, enabling closer monitoring, improved medical management, and potentially earlier interventions for high-risk patients. Future large-scale, prospective studies using standardized imaging protocols are necessary to confirm these findings and to assess whether MW-guided clinical pathways can enhance long-term outcomes after TAVR.

## CONCLUSIONS

Our review with a limited number of studies shows that MW indices may have a role in predicting patient outcomes after TAVR. GWI in particular may be a predictor of adverse outcomes, especially mortality. However, evidence is limited and scarce for GCW and GWE, while GWW may not be a predictor of outcomes.
